# Genome-Wide Linkage Mapping of QTL for Adult-Plant Resistance to Stripe Rust in a Chinese Wheat Population Lantian 25 × Huixianhong

**DOI:** 10.3390/plants14162571

**Published:** 2025-08-18

**Authors:** Fangping Yang, Yamei Wang, Ling Wu, Ying Guo, Xiuyan Liu, Hongmei Wang, Xueting Zhang, Kaili Ren, Bin Bai, Zongbing Zhan, Jindong Liu

**Affiliations:** 1 Wheat Research Institute, Gansu Academy of Agricultural Sciences, Lanzhou 730070, China; gy0931@163.com (Y.G.); xiuyanliu1124@163.com (X.L.); zhxueting0225@163.com (X.Z.); bbaigau@163.com (B.B.); zzb@gsagr.ac.cn (Z.Z.); 2 College of Life Sciences, Langfang Normal University, Langfang 065000, China; wangyamei@lfnu.edu.cn; 3 Crop Research Institute, Sichuan Academy of Agricultural Sciences, Chengdu 610066, China; wuzero50@163.com; 4 Institute of Biotechnology, Gansu Academy of Agricultural Sciences, Lanzhou 730070, China; wanghm@gsagr.ac.cn; 5 Vegetable Research Institute, Gansu Academy of Agricultural Sciences, Lanzhou 730070, China; renkaili66@126.com; 6 Institute of Crop Science, National Wheat Improvement Center, Chinese Academy of Agricultural Sciences, Beijing 100081, China

**Keywords:** *Puccinia striiformis*, quantitative trait loci, single-nucleotide polymorphism (SNP), *Triticum aestivum*

## Abstract

Stripe rust, caused by *Puccinia striiformis* f. sp. *tritici* (Pst), represents a major global threat to wheat (*Triticum aestivum*. L). Planting varieties with adult-plant resistance (APR) is an effective approach for long-term management of this disease. The Chinese winter wheat variety Lantian 25 exhibits moderate-to-high APR against stripe rust under field conditions. To investigate the genetic basis of APR in Lantian 25, a set of 219 F_6_ recombinant inbred lines (RILs) was created from a cross between Lantian 25 (resistant parent) and Huixianhong (susceptible parent). These RILs were assessed for maximum disease severity (MDS) in Pixian of Sichuan and Qingshui of Gansu over the 2020–2021 and 2021–2022 growing seasons, resulting in data from four different environments. Genotyping was performed on these lines and their parents using the wheat Illumina 50K single-nucleotide polymorphism (SNP) arrays. Composite interval mapping (CIM) identified six quantitative trait loci (QTL), named *QYr.gaas-2BS*, *QYr.gaas-2BL*, *QYr.gaas-2DS*, *QYr.gaas-2DL*, *QYr.gaas-3BS* and *QYr.gaas-4BL*, which were consistently found across two or more environments and explained 4.8–12.0% of the phenotypic variation. Of these, *QYr.gaas-2BL*, *QYr.gaas-2DS*, and *QYr.gaas-3BS* overlapped with previous studies, whereas *QYr.gaas-2BS*, *QYr.gaas-2DS*, and *QYr.gaas-4BL* might be novel. All the resistance alleles for these QTL originated from Lantian 25. Furthermore, four kompetitive allele-specific PCR (KASP) markers, *Kasp_2BS_YR* (*QYr.gaas-2BS*), *Kasp_2BL_YR* (*QYr.gaas-2BL*), *Kasp_2DS_YR* (*QYr.gaas-2DS*) and *Kasp_2DL_YR* (*QYr.gaas-2DL*), were developed and validated in 110 wheat diverse accessions. Additionally, we identified seven candidate genes linked to stripe rust resistance, including disease resistance protein RGA2, serine/threonine-protein kinase, F-box family proteins, leucine-rich repeat family proteins, and E3 ubiquitin-protein ligases. These QTL, along with their associated KASP markers, hold promise for enhancing stripe rust resistance in wheat breeding programs.

## 1. Introduction

Stripe rust (yellow rust, YR), caused by *Puccinia striiformis* f. sp. *tritici* (Pst), is a highly damaging fungal disease affecting common wheat (*Triticum aestivum*). It predominantly thrives in temperate, medium-altitude, and maritime wheat-growing regions [1]. Yield losses attributed to YR range from 10 to 70%, with over 20 significant epidemics reported globally [2]. In recent years, wheat YR has affected approximately 4.2 million hectares of farmland annually, causing significant economic losses to the wheat cultivation industry in the southwestern and northwestern regions of China [2]. While fungicides offer control, their effectiveness is constrained by management and financial limitations. Consequently, resistant cultivars represent an economically viable and environmentally sustainable strategy against this disease [2,3].

Resistance to YR is broadly classified into all-stage resistance and adult-plant resistance (APR) [1,4,5]. All-stage resistance typically involves major, race-specific genes inherited qualitatively [6], but tends to be short-lived due to the rapid adaptation of new pathogen races [7]. In contrast, APR, conferred by minor, typically race non-specific genes inherited quantitatively, offers more durable resistance [5]. Combining multiple APR genes can provide effective, lasting resistance, such as *Yr18* combined with additional minor genes, which has protected against YR for over 80 years in various countries including China [8].

Currently, over 90 wheat YR resistance genes at nearly 70 loci have been formally cataloged, predominantly race-specific, with many in China having been overcome by new pathogen races [9]. Among these, nineteen APR genes at various loci have been identified, some with pleiotropic effects conferring resistance to other diseases [4], namely, *Yr18* (7DS) [10], *Yr29* (1BL) [11], *Yr30* (3BS) [12], *Yr34* (5AL) [13], *Yr46* (4DL) [14], *Yr48* (5AL) [15], *Yr49* (3DS) [16], *Yr54* (2DL) [17], *Yr56* (2AS) [18], *Yr58* (3BS) [19], *Yr60* (4AL) [20], *Yr68* (4BL) [21], *Yr71* (3DL) [22], *Yr75* (7AL) [23], *Yr77* (6DS) [24], *Yr78* (6BS) [25], *Yr80* (3BL) [26], *Yr83* (6RL) [27], and *Yr86* (2AL) [28,29,30], whereas *Yr36* (6BS) [31], *Yr39* (7BL) [32], *Yr52* (7BL) [33], *Yr59* (7BL) [34], *Yr62* (4BL) [35], and *Yr79* (7BL) [3] are high-temperature adult-plant (HTAP) resistance genes against stripe rust [10,11,12,13,14]. Additionally, *Yr18/Lr34/Sr57/Pm38* [10], *Yr29/Lr46/Sr58/Pm39* [11], *Yr30/Lr27/Sr2/Pm70* [12], and *Yr46/Lr67/Sr55/Pm46* [14] are pleiotropic genes which have been widely utilized in breeding programs alongside all-stage resistance genes such as *Yr5*, *Yr10*, *Yr15*, and *Yr24/Yr26/YrCH42* [19,26,36,37,38,39,40,41,42,43]. To date, the successfully cloned genes include *Yr5* [36], *Yr10* [37], *Yr15* [38], *YrU1* [39], *Yr27* [40], *Yr28* [41], *Yr36* [19], *Yr18* [26], *Yr46* [42,43], and *YrNAM* [44].

Over the past two decades, numerous quantitative trait loci (QTL) for YR resistance have been identified across 49 chromosomal regions [30]. Molecular markers, particularly SNP arrays, facilitate combining APR and all-stage resistance in breeding programs. However, challenges such as low polymorphism and the vast genome of common wheat limit mapping resolution. High-density linkage maps constructed using SNPs enable precise QTL analysis and identification of candidate genes.

Lantian 25, a semi-dwarf winter wheat variety, exemplifies moderate-to-high resistance to YR at maturity while being susceptible at the seedling stage, demonstrating a typical APR response. This study aims to identify APR QTL in a Lantian 25 × Huixianhong recombinant inbred line (RIL) population using SNPs and to develop markers for wheat YR resistance breeding.

## 2. Results

### 2.1. Phenotypic Evaluation

**Figure A1:**
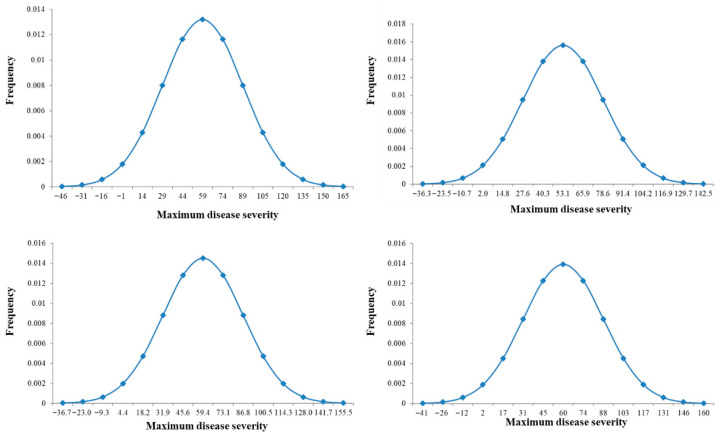
Frequency distributions for stripe rust responses in the Lantian 25/Huixianhong RIL population.

The moderately resistant parent, Lantian 25, demonstrated a mean maximum disease severity (MDS) of 20.3%, 25.6%, 20.7%, and 20.0% in Pixian 2021, Qingshui 2021, Pixian 2022, and Qingshui 2022, respectively. In contrast, the susceptible parent, Huixianhong, displayed a mean MDS of 90.5%, 100.0%, 100.0%, and 95.0% across the four environments, respectively (Figure A1). For the 219 RILs, the mean MDS were 59.7%, 59.3%, 59.4%, and 53.1% in Pixian 2021, Qingshui 2021, Pixian 2022, and Qingshui 2022, respectively. The MDS ranges for these environments were 5.0–100.0%, 1.0–100.0%, 3.0–100.0%, and 0–100.0%, respectively, indicating substantial polygenic variation in the population. Statistical analysis revealed significant correlations (*r* = 0.57–0.93; *p* < 0.01) for MDS across environments.

Analysis of variance (ANOVA) for MDS revealed highly significant differences (*p* < 0.01) among RILs, environments, and line × environment interactions (Table A1), indicating that stripe rust resistance is influenced by both genetic and environmental factors. The observed distribution of MDS and the significant genotype × environment interactions emphasize the quantitative nature of adult-plant resistance to stripe rust. The *H*_b_^2^ for MDS was calculated as 0.72, suggests that a considerable proportion of the phenotypic variance is attributable to genetic factors, making this population suitable for QTL mapping and further genetic studies of stripe rust resistance.

### 2.2. QTL for APR to Stripe Rust

**Figure A2:**
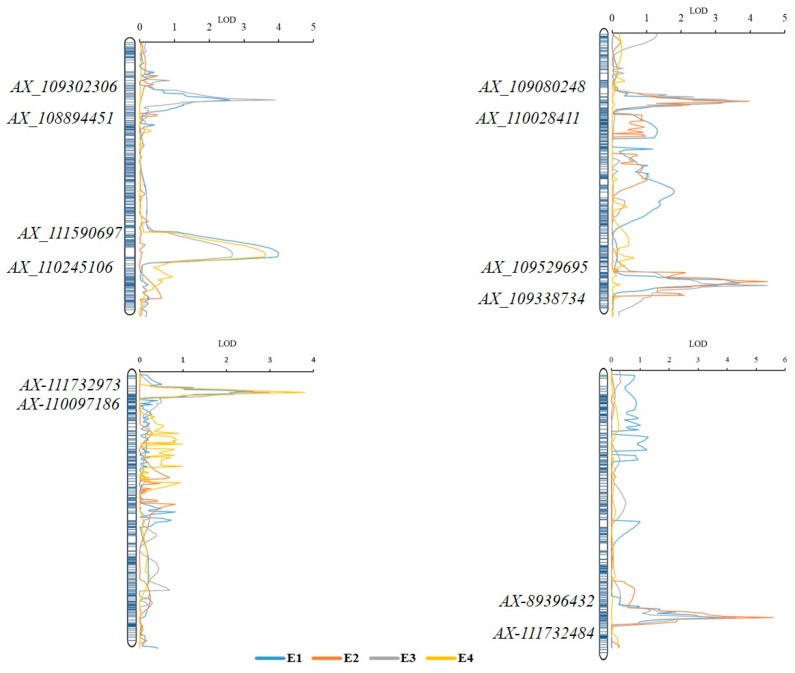
QTL and corresponding LOD curves identified in this study.

Six QTL for YR resistance were identified in this study, namely, *Qyr.gaas-2BS*, *QYr.gaas-2BL*, *QYr.gaas-2DS*, *QYr.gaas-2DL*, *QYr.gaas-3BS*, and *QYr.gaas-4BL* (Table 1). All the resistance alleles were contributed by Lantian 25. A major and consistent QTL, *QYr.gaas-4BL*, was flanked by *AX-89396432* and *AX-111732484* at 665.4–667.9 Mb in Pixian2020, Qingshui 2020, and Pixian 2021, and explained 8.6–12.0% of the phenotypic variances (PVE). *QYr.gaas-2BS*, located at the interval of *AX_109302306* (96.1 Mb) and *AX_108894451* (102.8 Mb), in Pixian2020 and Pixian2021, explained 7.5–8.6% of the PVE. *QYr.gaas-2BL*, closely linked with *AX_111590697* (672.3 Mb) and *AX_110245106* (697.9 Mb) in Pixian2020, Pixian 2021, and Qingshui 2021, respectively, explained 4.8–8.5% of the PVE. Two loci for YR resistance were identified on the 2D chromosome. *QYr.gaas-2DS*, between *AX_109080248* and *AX_110028411* and located at the 96.1–102.8 Mb in Pixian2020, Pixian 2021, and Qingshui 2021, respectively, explained 7.5–8.6% of the PVE. *QYr.gaas-2DL*, between *AX_109529695* and *AX_109338734* and located at the 596.0–608.6 Mb in Pixian2020, Pixian 2021 and Qingshui 2020, respectively, explained 5.2–9.6% of the PVE. *QYr.gaas-3BS*, between *AX-111732973* and *AX-110097186* and located at the 2.49–8.04 Mb on chromosome 3BS in Pixian2020, Pixian 2021, and Qingshui 2021, respectively, explained 4.9–8.5% of the PVE (Figure A2).

### 2.3. Candidate Gene Identification

A total of 707 annotated genes were present in the QTL regions for wheat stripe rust identified in this study and are listed in Table S1. A total of seven candidate genes were selected from the QTL regions for wheat stripe rust identified in this study, primarily involved in biological metabolism of disease resistance response, signal transformation, and the ubiquitin pathway (Table 2). Among these candidates, *TraesCS2B02G479500* for *QYr.gaas-2BL* encoded the disease resistance protein family. *TraesCS2B02G487600* for *QYr.gaas-2BL* encoded the disease resistance protein RGA2. For *QYr.gaas-2DS* and *QYr.gaas-2DL*, *TraesCS2D02G154700* and *TraesCS2D02G505900* were selected as the candidate genes and encoded the serine/threonine-protein kinase. For *QYr.gaas-4BL* and *QYr.gaas-2BS*, *TraesCS4B02G391600* and *TraesCS2B02G130600* encoded the F-box family proteins. One candidate gene for *QYr.gaas-3BS* was selected, *TraesCS3B02G013100*, and encoded the glycosyltransferase.

On the right is the expression level of the candidate genes, with darker colors indicating higher expression levels. On the left is information about the organs where the genes are expressed and the physiological and biochemical pathways they are involved in. The expression data are sourced from https://www.wheat-expression.com/ (accessed on 1 June 2025).

### 2.4. Development and Validation of Kompetitive Allele-Specific PCR (KASP) Markers

Efforts were made to develop KASP markers for all six identified QTL: *QYr.gaas-2BS*, *QYr.gaas-2BL*, *QYr.gaas-2DS*, *QYr.gaas-2DL*, *QYr.gaas-3BS*, and *QYr.gaas-4BL*. However, attempts to develop KASP markers for *QYr.gaas-3BS* and *QYr.gaas-4BL* were unsuccessful in effectively discriminating between parental genotypes within the RIL population, resulting in inconclusive outcomes (Table A2). Consequently, four KASP markers were successfully developed based on tightly linked KASP markers: *Kasp_2BS_YR* (*QYr.gaas-2BS*, *99.7* Mb), *Kasp_2BL_YR* (*QYr.gaas-2BL*, 697.7 Mb), *Kasp_2DS_YR* (*QYr.gaas-2DS*, 12.7 Mb), and *Kasp_2BL_YR* (*QYr.gaas-2BL*, *602.5* Mb). To validate the efficacy of these markers, a diverse panel with 111 cultivars was employed. For *Kasp_2BS_YR*, the favorable allele (CC) was present in 75 accessions, exhibiting a mean MDS of 38.0%. In contrast, the unfavorable allele (TT) was found in 32 accessions, with a mean MDS of 45.9%. This difference was statistically significant at *p* = 0.05 (Table 3 and Table A3). For *Kasp_2BL_YR*, the favorable allele (CC) was present in 69.4% of the cultivars, exhibiting a mean MDS of 39.1%. In contrast, the unfavorable allele (GG) was found in 27.9% of cultivars, with a mean MDS of 43.9%. This difference was statistically significant at *p* = 0.05 (Table A2). Similarly, for *Kasp_2DS_YR*, the favorable allele (GG) was observed in 55.0% of cultivars, demonstrating a mean MDS of 37.8%. The unfavorable allele (CC) was present in 63 cultivars, with a mean MDS of 38.3%. For *Kasp_2DL_YR*, the favorable allele (CC) was present in 63 of the cultivars, exhibiting a mean MDS of 38.3%. In contrast, the unfavorable allele (TT) was found in 48 cultivars, with a mean MDS of 43.7%. This difference was statistically significant at *p* = 0.05 (Table A2). This distinction was also statistically significant at *p* = 0.05.

## 3. Discussion

To date, over 350 wheat stripe rust resistance QTL or genes have been identified from wheat and related species through linkage analysis or association mapping methodologies. In the study, *QYr.gaas-2BS* (97.9 Mb) and *QYr.gaas-2BL* (676.8 Mb) were identified. Stripe rust resistance genes, including *Yr5/YrSP* (739.4 Mb), *Yr7* (660.0 Mb), *QYr.sicau-2B.1* (732.1 Mb), *QYr.sicau-2B.2* (799.5 Mb), *QYr.sicau-2B* (759.0 Mb), *QYr.caas-2BL.2* (685.8 Mb), *QYr.spa-2B.1* (280.9 Mb), *QYrqn.nwafu-2BL* (674.0 Mb), *QYr.nwafu-2BL* (796.8 Mb), *QYraq.cau-2BL* (671.0 Mb), *QYr.inra-2BL* (615.8–621.0 Mb), and *QYr.caas-2BL* (693.7–733.2 Mb), were identified on chromosome 2BS and 2BL [45,46,47,48,49,50,51,52]. Among them, *QYrqn.nwafu-2BL* (674.0 Mb) and *QYraq.cau-2BL* (671.0 Mb) overlapped with *QYr.gaas-2BL* identified in our study. However, no overlap was observed between *QYr.gaas-2BS* and previously identified loci on chromosome 2B. Thus, *QYr.gaas-2BS* might be novel.

In the present study, *QYr.gaas-2DS* and *QYr.gaas-2DL* were localized to the distal region of chromosome 2DS and 2DL, with the peak marker positioned between 96.1–102.8 Mb and 596.0–608.6 Mb. To date, six stripe rust resistance genes have been identified on chromosome 2D: *Yr8*, *Yr16*, *Yr54*, *Yr55*, *Yr37*, and *YrCK* [45,53,54,55]. The APR gene *Yr16* and *QYr.inra-2DS* have been mapped to the centromeric region of chromosome 2D [46], indicating that these loci are distinct from *QYr.gaas-2DS*. Furthermore, Ren et al. [47] reported a QTL on chromosome 2D flanked by the SSR markers *Xgwm539* (513.1 Mb) and *Xcfd44* (608.6 Mb), which overlapped with *QYr.gaas-2DL* identified in this study. Several other QTL have been mapped to various positions on chromosome 2D: *QYr.ufs-2D* (72.6 Mb) [56], *QYr.caas-2DS* (12.3–19.6 Mb) [54], *QYr.2DS* (19.6 Mb) [53], and *QYr.inra-2DS* (72.6 Mb) [23]. The distinct genomic location of *QYr.gaas-2DS* compared to previously reported QTL suggests that it may represent a novel locus conferring stripe rust resistance.

The 3B chromosome is rich in stripe rust resistance genes [57,58,59]. Over 15 loci for stripe rust have been identified distributed on the 3B chromosome [28,29,30]. Of these, *QYr.cimmyt-3BS* from Pavon76 [33], *QYr.inra-3BS* from Renan [21], *QYr.ucw-3BS* from UC110 [60], and *QRYr-3B* from Pastor have shown effective and stable resistance for stripe rust. Furthermore, the well-known adult-plant resistance gene for stripe rust, *Yr30*, is located on chromosome 3BS, overlapping with the locus identified in this study. Thus, we hypothesized that *QYr.gaas-3BS* might correspond to *Yr30*. To verify this, we genotyped Lantian 25 using the reported marker (*gwm533*) and confirmed that Lantian 25 carries *Yr30* [34]. This finding supports the conclusion that *QYr.gaas-3BS* is indeed *Yr30*.

*Yr18*, *Yr29*, *Yr30*, and *Yr46* have good resistance effects against stripe rust and are widely used in Chinese varieties, especially in the Huang-Huai winter wheat region and the southwestern wheat region. We conducted extensive testing for *Yr18*, *Yr29*, *Yr30*, and Yr46 in 2014 and have well-established primers and detection systems. However, only Yr30 was detected in Lantian25 and maybe *QYr.gaas-3BS*. Furthermore, *Yr18*, *Yr29* and Yr46 are located on chromosomes 7DS, 1BL, and 4DL, respectively. In this study, we detected eight loci related to wheat stripe rust resistance in Lantian25, located on chromosomes 2BS, 2BL, 2DS, 2DL, 3BS, and 4BL, none of which are on the same chromosomes as the three important stripe rust resistance genes mentioned above. In addition to *Yr18*, *Yr29*, *Yr30*, and *Yr46*, we also tested for *Yr17* and *Yr26*, but none were detected in Lantian25.

Nine stable and effective loci for stripe rust resistance were identified on chromosome 4BL [22,61,62,63,64], namely, *Qyr.wpg-4B.1*(531.1 Mb), *Yr62* (509.0–568.6 Mb) [22], *QYrhm.nwafu-4B* (523.4–568.6) [62], *QYr.caas-4BL* (509.0–544.6), *QYr.nwafu-4BL* (189.7–192 Mb), *QYr.nwafu-4BL* (640.1 Mb), *QPst.jic-4B* (610.6 Mb), *QPst.jic-4B* (592.6–622.3 Mb), and *QYr.sun-4B* (250.0 Mb) [19,26,41,42,43,65,66]. No overlap was identified between *QYr.gaas-4BL* (665.4–667.9 Mb) and the loci mentioned above [22,61,62,63,64,65]. Thus, *QYr.gaas-4BL* may be novel.

**Figure 1:**
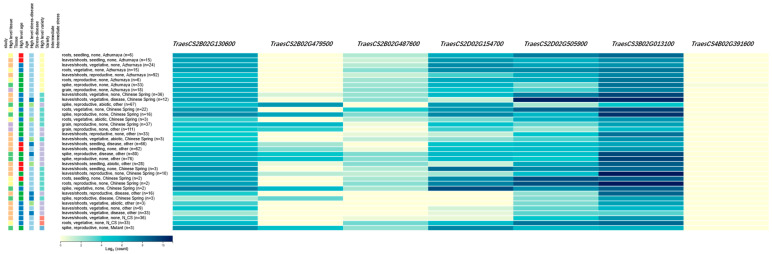
Expression patterns for the candidate genes identified in this study.

In total, seven candidate genes were selected based on the genetic interval, annotation, and expression patterns (Figure 1). *TraesCS2B02G479500* and *TraesCS2B02G487600* (*QYr.gaas-2BL*) encoded the disease resistance protein family. The majority of plant disease resistance genes encode disease resistance proteins, including leaf rust, stripe rust, or powdery mildew resistance [67,68], like *TaRPS2* for wheat stripe rust [66] and *TaRPP13* for powdery mildew [67]. In plants, the disease resistance protein family gene exhibits a higher degree of conservation. Previous studies have indicated that the wheat leaf rust resistance gene *Lr10* may function as a guardee, directly interacting with pathogen effectors, while RGA2 assumes the role of a guard. In other proposed models, the plant RGA2 gene recognizes pathogen effectors through indirect mechanisms, resulting in a more complex process [68,69]. Both *TraesCS2D02G154700* for *QYr.gaas-2DS* and *TraesCS2D02G505900* for *QYr.gaas-2DL* encoded the serine/threonine-protein kinase, which significantly associated with powdery mildew or septoria leaf blotch [70,71]. For *QYr.gaas-4BL* and *QYr.gaas-2BS*, *TraesCS4B02G391600* and *TraesCS2B02G130600* encoded the F-box family proteins, in response to various pathogens in the degradation machinery [72,73]. *TraesCS3B02G013100* for *QYr.gaas-3BS* was selected and encoded as the glycosyltransferase, which is annotated as a component of plant immune signaling pathways [74].

While conventional breeding methodologies have contributed significantly to the enhancement of disease resistance, the selection process remains time-intensive and relatively inefficient [75]. QTL identified across multiple environments offer promising potential for implementation in MAS strategies. The present investigation corroborates previous findings, indicating that the incorporation of 4–5 APR genes with minor-to-intermediate effects within a single line may confer enhanced levels of resistance to stripe rust. This observation underscores the potential for pyramiding multiple resistance loci to achieve durable and broad-spectrum resistance. KASP, a uniplex SNP genotyping platform that provides a cost-effective and scalable approach for applications requiring small-to-moderate marker numbers, such as MAS and QTL fine mapping. In this study, *Kasp_2BS_YR*, *Kasp_2BL_YR*, *Kasp_2DS_YR*, and *Kasp_2DL_YR* were successfully developed based on tightly linked SNP markers. These markers have demonstrated their utility as valuable tools for MAS in wheat breeding programs aimed at improving yellow rust resistance. By enabling rapid and accurate genotyping of resistance-associated loci, these markers facilitate the efficient introgression of beneficial alleles into elite germplasm. Furthermore, the ability to combine multiple resistance loci through MAS offers the potential to develop wheat cultivars with enhanced and durable resistance to stripe rust.

## 4. Materials and Methods

### 4.1. Germplasm Development and Characterization

A population of 219 F_2:6_ RILs was derived from the cross between Lantian 25 and Huixianhong. Both parental lines exhibited high susceptibility to the predominant Pst races CYR29, CYR31, CYR32, CYR33, and CYR34 at the seedling stage. However, Lantian 25 demonstrated moderate resistance at the adult-plant stage under field conditions. The RIL population was developed using the single seed descent method, with one randomly selected spike harvested per generation for advancement. Additionally, 110 wheat cultivars, predominantly from the Yellow-Huai Wheat Region, was utilized to validate the efficacy of KASP markers for stripe rust resistance.

### 4.2. Field Experimentation

The F_2:6_ RILs and their progenitors were evaluated for APR to YR at two locations: Pixian (30°05′ N, 102°54′ E) and Qingshui (34°05′ N, 104°35′ E). Trials were conducted during the 2020–2021 and 2021–2022 cropping seasons, providing data from four distinct environments. Both sites are recognized YR hotspots in China, offering optimal conditions for rust pathogen proliferation. The experimental design employed randomized complete blocks with two replicates per location. Each plot consisted of a single 1.5 m row, with 0.25 m inter-row spacing. About 50 seeds were sown per row. The highly susceptible cultivar Huixianhong was planted every tenth row as a disease spreader. To ensure sufficient inoculum pressure, border rows of cv. Huixianhong encircled the experimental plots at both locations.

Inoculations were performed at the jointing stage using a mixture of prevalent Chinese Pst races (CYR31, CYR32, CYR33, and CYR34) applied via spray method. Inoculations occurred around January 5 in Pixian and April 10 in Qingshui. Disease severity (DS) assessments commenced 12–18 days post-inoculation, once the susceptible control Huixianhong exhibited pronounced symptoms. DS was recorded weekly, typically 2–3 times, with the MDS selected for subsequent analysis and QTL mapping.

### 4.3. Genotyping and Genetic Map Construction and QTL Analysis

All 219 RILs were genotyped using the wheat 50K iSelect SNP array. SNPs with >20% missing data or minor allele frequency (MAF) <0.5 were excluded from further analysis according to Wen et al. [76]. The filtered SNPs were grouped into bin markers by IciMapping v4.2 (https://isbreeding.caas.cn/rj/qtllcmapping/294445.htm (accessed on 1 June 2025)) [77] and the linkage map was constructed by the regression mapping algorithm (JoinMap v4.0) (JoinMap v4.0). QTL analysis was conducted by inclusive composite interval mapping (ICIM) implemented in IciMapping 4.1 according to Liu et al. [78]. A logarithm of odds (LOD) threshold of 2.75 was established for QTL significance based on 1000 permutations at *p* = 0.05. Physical positions for SNPs were according to the IWGSC2.1.

MDS data from four environments across three cropping seasons were utilized for ANOVA conducted using IciMapping 4.1. The contributions of RILs and environments were assessed using PROC MIXED, with environments treated as fixed effects, while lines, line × environment interactions, and replicates nested within environments were considered random effects. Broad-sense heritability (*H*_2_^b^) for YR was calculated using the formula: *H*_2_^b^ = σ^2^_g_/(σ^2^_g_ + σ^2^_ge_/r + σ^2^_ε_/re). Of these, σ^2^_g_: genotypic; σ^2^_ge_: genotype × environment interaction; and σ^2^: residual error variances, respectively. The terms e and r denote the number of environments and replicates per environment, respectively.

### 4.4. KASP Marker Development and Candidate Gene Identification

SNPs were converted to KASPs using PolyMarker. Genotyping was performed using 384-well plates and analyzed on a PHERA starplus SNP platform. Genotype calling was conducted using KlusterCaller v1.0 software (LGC company, Londong, UK). All KASP markers were validated using the panel of 110 cultivars. The validation panel consisted of 111 wheat accessions, primarily from different wheat-growing regions in China, including modern cultivars, advanced lines, and landraces. All accessions were evaluated for stripe rust resistance during the 2014–2015 and 2015–2016 cropping seasons at the Pixian Experimental Station of the Sichuan Academy of Agricultural Sciences and the Gangu Qingshui Experimental Station of the Gansu Academy of Agricultural Sciences, as well as during the 2014–2015 cropping season at the Zhongliang Experimental Station of the Tianshui Institute of Agricultural Science in Gansu Province. These three locations are hotspots for stripe rust in China, with environmental conditions conducive to disease development. Field trials were conducted using a randomized complete block design with three replicates, three-row plots, 20 cm row spacing, and 1.5 m row length. Every 10th row was planted with the highly susceptible control cultivar Huixianhong. Inoculation was performed at the jointing stage by spraying a mixture of prevalent Chinese races, including CYR32, CYR33, and CYR34. MDS was recorded 18 to 20 days after flowering. The MDS from each environment and the mean values across the five environments for each accession were used for subsequent validation analysis. Differences in stripe rust severity between KASP marker classes were analyzed using Student’s *t*-test to determine statistical significance.

To elucidate potential candidate genes associated with stripe rust resistance QTL detected in the Lantian 25/Huixianhong RIL population, genes located at the confidence interval of each QTL were extracted from the wheat IWGSC v2.1. Genes were considered candidates if they contained SNPs in coding regions and were not annotated as hypothetical, transposon, or retrotransposon proteins.

## 5. Conclusions

This study elucidates the genetic basis of APR to stripe rust in Lantian 25. Six QTL, namely, *QYr.gaas-2BS*, *QYr.gaas-2BL*, *QYr.gaas-2DS*, *QYr.gaas-2DL*, *QYr.gaas-3BS*, and *QYr.gaas-4BL*, were consistently identified, explaining 4.8–12.0% of PVEs. Among these, *QYr.gaas-2BL*, *QYr.gaas-2DS*, and *QYr.gaas-4BL* overlap with previously reported loci, while *QYr.gaas-2BS*, *QYr.gaas-2DS*, and *QYr.gaas-4BL* represent potential novel loci. Notably, all resistance alleles originated from Lantian 25. The development of validated four KASP markers (*Kasp_2BS_YR, Kasp_2BL_YR, Kasp_2DS_YR*, and *Kasp_2DL_YR*) and identification of candidate genes (e.g., disease resistance proteins, kinases, and ubiquitin ligases) provide actionable tools for MAS breeding. These findings advance our understanding of APR mechanisms and offer robust genetic resources and molecular tools for enhancing durable stripe rust resistance in wheat breeding.

## Figures and Tables

**Table 1:** QTL for stripe rust resistance in the Lantian 25 × Huixianhong RIL population.

QTL	Environment	Genetic Interval	Start (Mb)	End (Mb)	LOD	PVE (%)	Add
*QYr.gaas-2BS*	E1, E3	*AX_109302306-* *A_108894451*	96.0	102.8	2.6–3.9	5.0–8.4	6.1–8.0
*QYr.gaas-2BL*	E1, E3, E4	*AX_111590697-* *AX_110245106*	672.3	697.7	2.5–4.0	4.8–8.5	6.3–8.1
*QYr.gaas-2DS*	E1, E2, E3	*AX_109080248-* *AX_110028411*	96.1	102.9	3.6–3.9	7.5–8.6	6.5–8.3
*QYr.gaas-2DL*	E1, E2, E3	*AX_109529695-* *AX_109338734*	596.0	608.6	2.7–4.5	5.2–9.6	6.3–8.3
*QYr.gaas-3BS*	E1, E3, E4	*AX-111732973-* *AX-110097186*	2.49	8.04	2.6–3.8	4.9–8.5	6.2–7.9
*QYr.gaas-4BL*	E1, E2, E3	*AX-89396432-* *AX-111732484*	665.4	667.9	3.9–5.6	8.6–12.0	7.5–10.2

**Table 2:** The candidate genes for RSA-related traits identified in the Doumai/Shi 4185 RIL population.

QTL	Candidate Gene	Start (Mb)	Annotation
*QYr.gaas-2BS*	*TraesCS2B02G130600*	97.9	F-box family proteins
*QYr.gaas-2BL*	*TraesCS2B02G479500*	676.8	Disease resistance protein family
*QYr.gaas-2BL*	*TraesCS2B02G487600*	683.7	Disease resistance protein RGA2
*QYr.gaas-2DS*	*TraesCS2D02G154700*	98.3	Serine/threonine-protein kinase
*QYr.gaas-2DL*	*TraesCS2D02G505900*	599.9	Serine/threonine-protein kinase
*QYr.gaas-3BS*	*TraesCS3B02G013100*	6.06	Glycosyltransferase
*QYr.gaas-4BL*	*TraesCS4B02G391600*	667.4	F-box family protein

**Table 3:** Effects of four developed KASP markers on stripe rust resistance in the natural population.

QTL	Genotype	Number of Lines	MDS (%)	*p*-Value
*Kasp_2B* *S_YR*	TT	32	45.9	0.030 *
	CC	75	38.0	
*Kasp_2B* *L_YR*	GG	31	43.9	0.039 *
	CC	77	39.1	
*Kasp_2D* *S_YR*	GG	61	37.8	0.043 *
	AA	49	44.2	
*Kasp_2D* *L_YR*	TT	48	43.7	0.022 *
	CC	63	38.3	

* indicate significant at *p* < 0.05.

## Data Availability

Data are contained within the article and Supplementary Materials.

## Supplementary Materials

The following supporting information can be downloaded at: https://www.mdpi.com/article/10.3390/plants14162571/s1, Table S1: The higher confidence genes located in the QTL for stripe rust resistance in this study.

## Abbreviations

APR	Adult-plant resistance
RILs	Recombinant inbred lines
SNP	Single-nucleotide polymorphism
SR	Stripe rust
QTL	Quantitative trait loci
KASP	Kompetitive allele-specific PCR
MAS	Marker-assisted selection
PVE	Phenotypic variance explained
YR	Yellow rust

## Appendix A

**Table A1:** Analysis of variance of the maximum disease severity for stripe rust in the Lantian 25/Huixianhong RIL population.

Source of Variance	Df	Mean Square	*F* Value
Replicate (environment)	2	1571	8.8 *
Environment	4	38,028	212.0 *
Line	218	3125	17.4 *
Line × Environment	1088	311	1.7 *
Error	1654	179	

* Significant at *p* < 0.05.

**Table A2:** The KASP primers for the four markers developed in this study.

Kasp Marker	Prime	Sequence
*Kasp_2B* *S_Y*	FAX	GAAGGTGACCAAGTTCATGCTtgCtagtgaaatcaagtgtatgatA
	HEM	GAAGGTCGGAGTCAACGGATTtgCtagtgaaatcaagtgtatgatG
	Common	
*Kasp_2BL_YR*	FAX	GAAGGTGACCAAGTTCATGCTGGGTAGCATTGATCTAAACTCCTG
	HEM	GAAGGTCGGAGTCAACGGATTGGGTAGCATTGATCTAAACTCCTC
	Common	ctgagcgactgtttcttattgtT
*Kasp_2DS_YR*	FAX	GAAGGTGACCAAGTTCATGCTCTACAAACATGGTTCACATTGAC
	HEM	GAAGGTCGGAGTCAACGGATTACTACAAACATGGTTCACATTGAT
	Common	TGCATCATCTCCACCGGAAC
*Kasp_2* *DL_Y*	FAX	GAAGGTGACCAAGTTCATGCTgttctcgatctgtatgctgaagtA
	HEM	GAAGGTCGGAGTCAACGGATTgttctcgatctgtatgctgaagtG
	Common	agatgactttccagatgtagggA

**Table A3:** The genotype and stripe rust responses for the 111 wheat accessions by the four developed KASP markers.

Name	Origin	MDS (%)	*Kasp_2BS_YR*	*Kasp_2BL_YR*	*Kasp_2DS_YR*	*Kasp_2DL_YR*
Wheatear	USA	33.2	CC	CC	GG	CC
11CA40	Beijing	21.8	CC	CC	AA	CC
12CA29	Beijing	64.3	CC	CC	GG	CC
CA0548	Beijing	83.3	CC	CC	AA	TT
CA0816	Beijing	22.3	CC	CC	GG	CC
CA0958	Beijing	59.7	CC	GG	AA	TT
CA1055	Beijing	60.3	TT	GG	GG	CC
CA1090	Beijing	69.5	TT	GG	AA	TT
CA1119	Beijing	30.4	TT	GG	GG	CC
CA1133	Beijing	30.7	CC	CC	GG	CC
CA9722	Beijing	61.4	CC	CC	AA	TT
Jing411	Beijing	27.8	CC	CC	GG	CC
Jing9428	Beijing	68.3	CC	CC	AA	TT
Zhongmai175	Beijing	22.3	CC	GG	AA	TT
Zhongmai415	Beijing	74.6	CC	CC	GG	CC
Zhongyou9507	Beijing	50.5	CC	CC	AA	TT
Holdfast	Britain	18.5	CC	CC	GG	CC
00127-2-2	Gansu	7.9	CC	CC	GG	CC
863-13	Gansu	19.4	CC	CC	GG	CC
Baigetiao	Gansu	20.1	CC	CC	GG	TT
Baimangmai1	Gansu	24	TT	GG	GG	CC
CP07-9-1-1-2F6	Gansu	41.3	CC	CC	GG	CC
Dabaijiankou	Gansu	21.6	CC	GG	AA	TT
Damai1	Gansu	18.9	CC	CC	GG	CC
Hangxuan2	Gansu	25.8	CC	CC	GG	CC
Honghuomai	Gansu	33	CC	CC	GG	CC
Honglaomangmai	Gansu	43.6	TT	CC	GG	TT
Huomaizi	Gansu	31.5	CC	CC	AA	TT
Jinhuangmai	Gansu	24.4	TT	GG	GG	CC
Lanhangxuan122	Gansu	34.2	CC	CC	AA	TT
Lantian 25	Gansu	36.4	TT	GG	AA	TT
Lantian26	Gansu	10.3	CC	CC	GG	CC
Longdonghong	Gansu	23.8	CC	CC	GG	CC
Longjian103	Gansu	32.3	CC	CC	AA	TT
Longjian196	Gansu	26	CC	CC	GG	CC
Longjian301	Gansu	59.9	CC	CC	GG	CC
Pingliang45	Gansu	37.5	CG	CG	GG	CC
Tutoumai	Gansu	29.5	CC	CC	AA	TT
Xiaobeijiekou	Gansu	23.7	CC	CC	GG	CC
Xifeng20	Gansu	56.5	CC	CC	AA	CC
Youmangmazhamai	Gansu	47.3	CC	CC	GG	CC
Hengguan33	Hebei	45	CC	CC	AA	TT
Shijiazhuang8	Hebei	57.1	CC	GG	GG	CC
Ag303-29	Henan	39.8	TT	GG	AA	TT
Ag303-8	Henan	45	GC	GC	GG	CC
Ag311-8	Henan	47.1	CC	CC	AA	TT
Aikang58	Henan	58.2	CC	CC	GG	CC
Bainong64	Henan	37.5	CC	CC	AA	TT
C40468	Henan	46.7	TT	CC	GG	CC
C40469	Henan	51.9	TT	CC	AA	CC
C40470	Henan	54	TT	GG	GG	CC
C40472	Henan	55.3	CC	CC	AA	TT
C40473	Henan	48.4	CC	GG	GG	CC
C40477	Henan	38.2	CC	CC	GG	CC
C40481	Henan	46.1	TT	CC	AA	TT
C40485	Henan	24.1	CC	CC	GG	CC
C50835	Henan	42.1	TT	GG	AA	TT
C50837	Henan	26.6	TT	GG	AA	TT
C50843	Henan	39.2	CC	CC	GG	CC
C50845	Henan	25.2	TT	GG	GG	CC
C50850	Henan	52.5	CC	CC	AA	TT
C50851	Henan	51.3	CC	CC	AA	TT
C50855	Henan	53.6	CC	CC	GG	CC
C50865	Henan	39.3	GC	GC	AG	CC
He106	Henan	31	CC	CC	GG	CC
He109	Henan	45.6	TT	CC	AA	TT
He12	Henan	18.8	CC	CC	GG	CC
He120	Henan	25.8	CC	CC	AA	TT
He18	Henan	31.5	CC	CC	AA	TT
He193	Henan	19	CC	CC	GG	CC
He2	Henan	47.3	TT	CC	AA	TT
He222	Henan	26.6	CC	CC	AA	TT
He231	Henan	33.9	TT	GG	AA	TT
He244	Henan	19.4	CC	CC	GG	CC
He256	Henan	48.7	CC	CC	GG	CC
He3	Henan	30.3	TT	GG	AA	TT
He48	Henan	30.9	TT	GG	GG	CC
He53	Henan	38.5	TT	GG	AA	TT
He7	Henan	28.6	CC	CC	GG	CC
He87	Henan	27.8	CC	CC	AA	TT
Pingyuan50	Henan	11.1	CC	CC	GG	CC
Zheng9023	Henan	49.3	CC	CC	AA	TT
Zhengmai366	Henan	29.4	CC	CC	GG	CC
Zhongmai871	Henan	36.6	CC	CC	AA	TT
Zhongmai875	Henan	35.5	CC	GG	AA	TT
Zhongmai895	Henan	34.7	CC	CC	AA	TT
Zhoumai13	Henan	45.8	TT	CC	AA	TT
Zhoumai16	Henan	41.2	CC	CC	GG	CC
Zhoumai18	Henan	22.8	CC	CC	GG	CC
Libellula	Italy	19.3	TT	GG	AA	TT
Pascal	Italy	7.1	CC	CC	GG	CC
Abbondanza	Italy	64.2	TT	GG	GG	CC
Funo	Italy	8.6	CC	CC	AA	TT
Ningdong10	Ningxia	25	CC	CC	GG	CC
Ningdong10	Ningxia	25	CC	CC	GG	CC
Ningdong11	Ningxia	83.2	CC	CC	AA	TT
Lovrin10	Romania	69	TT	GG	GG	CC
Changwu131	Shaanxi	52.9	CC	CC	GG	CC
Xiaoyan6	Shaanxi	75.9	CC	CC	AA	TT
Jimai19	Shandong	46	TT	GG	GG	CC
Jimai22	Shandong	36.6	TT	GG	AA	TT
Jinan13	Shandong	74.2	CC	CC	GG	CC
Jinan17	Shandong	59.8	CC	CC	GG	CC
Liangxing66	Shandong	52.3	TT	GG	AA	TT
Linmai4	Shandong	51.8	TT	GG	AA	TT
Lumai21	Shandong	72.5	TT	CC	GG	CC
Weimai8	Shandong	60.4	CC	CC	GG	CC
Yannong19	Shandong	90.9	TT	GG	AA	TT
ChineseSpring	Sichuan	34.4	CC	CC	GG	CC
Chuanmai107	Sichuan	62.1	CG	CG	AA	TT
Fan6		75.9	TT	GG	GG	CC
